# Association between Chronotype, Physical Activity and Sedentary Behaviour: A Systematic Review

**DOI:** 10.3390/ijerph19159646

**Published:** 2022-08-05

**Authors:** Nuria Sempere-Rubio, Mariam Aguas, Raquel Faubel

**Affiliations:** 1 Clinical Biomechanics Research Unit (UBIC), Department of Physiotherapy, Universitat de València, Gasco Oliag 5, 46010 Valencia, Spain; 2 Gastroenterology Department, La Fe University and Polytechnic Hospital, 46026 Valencia, Spain; 3 Health Research Institute La Fe, Avenida Fernando Abril Martorell, 106, 46026 Valencia, Spain; 4 Joint Research Unit in ICT Applied to Reengineering Socio-Sanitary Process, IIS La Fe—Universitat Politècnica de València, 46026 Valencia, Spain; 5 PTinMOTION—Physiotherapy in Motion Multispeciality Research Group, Department of Physiotherapy, Universitat de València, Gasco Oliag 5, 46010 Valencia, Spain

**Keywords:** physical activity, sedentary behaviour, chronotype, morningness

## Abstract

Background: The aim of this systematic review is to compile and assess the scientific evidence about the relationship between chronotypes and physical activity (PA)**.** Methods: A systematic review was executed using a structured electronic search in PubMED, Cochrane Library, PsycInfo and Trip Database. The searches employed keywords such as chronotype, sleep, acrophase, chronotype preference, morningness, physical activity and sedentary, using MeSH terms. JBI critical tools were used to appraise methodological aspects. Results: This systematic review includes 23 studies and a total of 505,375 participants. The results show that evening chronotypes are associated with less PA and more time in sedentary activities. It occurs independently of the instruments used to collect information about chronotype and PA. Nevertheless, this association could be mitigated in young populations and university stages. Conclusions: The chronotypes are clearly associated with the PA level and the sedentary behaviour, especially in the population over their mid-twenties. Evening chronotypes are associated with less PA and more time in sedentary activities compared to morning chronotypes.

## 1. Introduction

Physical activity (PA) is a modifiable lifestyle factor contributing to the prevention and treatment of non-communicable diseases, and it also helps to improve health outcomes as quality of life and well-being. The World Health Organization (WHO) defines PA as “any bodily movement produced by skeletal muscles that require an expenditure of energy [1]”. In addition, following the perspective of the planetary health, the societies that consume fewer fossil fuels have cleaner air, safer mobility and therefore more active environment spaces [2]. In fact, physical inactivity is, currently, one of the more relevant global health issues generating a growing concern. Multiple opportunities for PA could be found by integrating PA into the settings in which people live, work and play. For instance, being more active at work, sports, active play, recreation and, especially, opting for walking and cycling as means of transportation enables the engagement in regular PA on a daily basis. PA is important in all ages from early childhood, adolescents, adults and older adults to enable healthy and active ageing. Nevertheless, the percentage of the world’s population that does not reach the minimum levels of PA recommendations is still high: 23.3% of the global population in 2010, and 27.5% in 2016 [3,4]. Worldwide, one out of four adults is not active enough [1].

Sleep is another modifiable lifestyle considered as a complex and multidimensional factor, including some distinct aspects such as sleep duration, sleep quality, chronotype and sleep timing. Different sleep-wake patterns exist in humans, showing wide inter-individual differences in the sleep and wake-up timings and preferences for morningness–eveningness [5]. According to the morning-eveningness questionnaire (MEQ), three chronotypes have been identified—morningness chronotype (MC), intermediate (IC) and eveningness (EC)—and are based on peak times of the day according to one’s circadian rhythm [6].

Referring to previous studies, a more evening chronotype seems to be associated to different health outcomes [7] and a high percentage of obesity [8], mental health issues [9,10,11,12], respiratory diseases, type 2 diabetes and hypertension [13,14]. Likewise, the chronotype seems to be associated with several lifestyle factors, such as eating timing [8,15], tobacco usage [16,17], alcohol consumption [18] and PA [19]. Some previous reviews have analysed the relationship between chronotype and athletic performance and the psychophysiological responses to PA [20] or heart rate variability [21]. Nevertheless, more evidence is still needed about the relationship between the chronotype and the PA level. The aim of this systematic review is to compile and assess the scientific evidence about the relationship between chronotypes, physical activity and sedentary behaviour.

## 2. Materials and Methods

This study follows the guidelines of the Preferred Reporting Items for Systematic Reviews and Meta-Analyses (PRISMA) [22] and it was registered at the International Prospective Register of Systematic Reviews (CRD42022331959).

### 2.1. Bibliographic Search

A systematic review was executed using a structured electronic search following the procedure proposed by PRISMA in PubMED, Cochrane Library, PsycInfo and Trip Database. The searches employed keywords such as chronotype, sleep, acrophase, chronotype preference, morningness, physical activity and sedentary, using MeSH terms (Supplementary Table S1). A manual search was also performed, including the references of the articles found and the related articles.

### 2.2. Selection Criteria

The articles published during the last 10 years (March 2012–March 2022 both included) assessing the relationship between chronotype and PA in humans were included, in any language and any study design. Concerning exclusion criteria, studies conducted on animal models, those performed on participants under the age of 18, systematic reviews and studies without results (e.g., protocols) were excluded. Automation tools were not employed for screening. A PECO approach (Patients, Exposure, Comparison and Outcome) was used as inclusion and exclusion criteria, which were assessed by the study team [23]. All the identified articles were independently analysed by at least two researchers from the present study, and the final selection of the articles to be included was made by consensus.

### 2.3. Assessment of Methodological Quality

For the quality assessment, the “Standardized instruments from the Joanna Briggs Institute System for the Unified Management, Assessment and Review of Information” (JBI SUMARI) checklist was used to report and critically appraise the methodological aspects of included studies [24]. These instruments included the JBI Critical Appraisal Checklist for Comparable Cohort and the JBI Critical Appraisal Checklist for Cross-sectional Studies, chosen accordingly to the study design [25].

### 2.4. Data Extraction

A data extraction form to collect data on the model type was used. The information that was extracted from each study related to the objective of the study and the year of publication, the country of implementation, the characteristics of the participants (age, sex, chronic diseases and other relevant information about the population), tools for collecting information on chronotype and PA and results for each included study. Two different reviewers selected studies, rated methodological quality and extracted data independently. If there were any disagreements between both investigators, a third independent researcher determined inclusion/exclusion. 

## 3. Results

**Figure 1:**
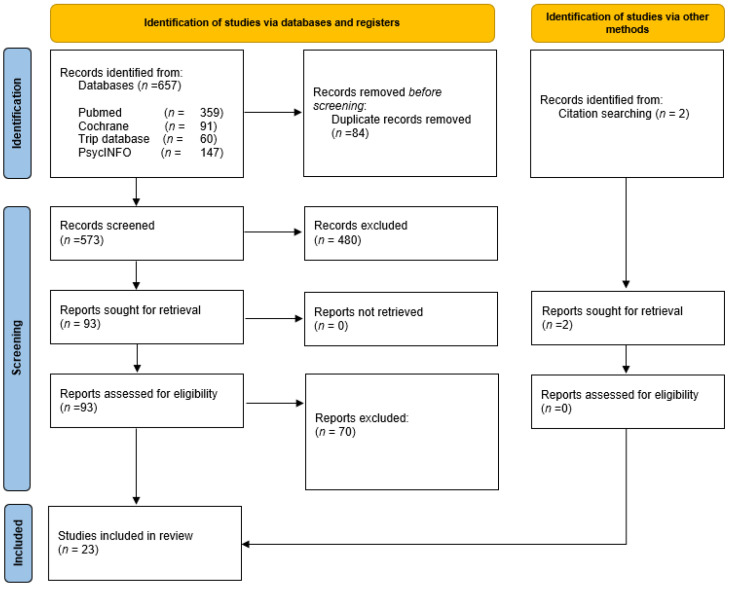
Flowchart of study selection process.

As shown in the PRISMA flow diagram (Figure 1), after the initial search, 657 articles were identified, of which 564 were eliminated after reading the title and summary and eliminating duplicates. Of the 93 remaining, after a critical reading of the full text, 71 other articles were rejected. Finally, 23 studies fitting the selection criteria were selected to be included in the systematic review.

Concerning the quality appraisal (Supplementary Tables S2 and S3), 90% of the included cross-sectional studies accomplished seven of the eight criteria. Longitudinal studies were over 70% of the items achieved and two of them accomplished 100% of the criteria.

### 3.1. Characteristics of the Included Studies

The characteristics of the 23 studies included are detailed in Table 1 and Table 2. These 23 studies were conducted in 14 different countries: USA (4 studies), UK (3 studies) and Korea, Brazil, Finland and Spain with 2 studies. The other eight countries contributed with one study: Italy, France, Switzerland, Czech Republic, Hungary, Turkey, Peru and China.

**Figure 2:**
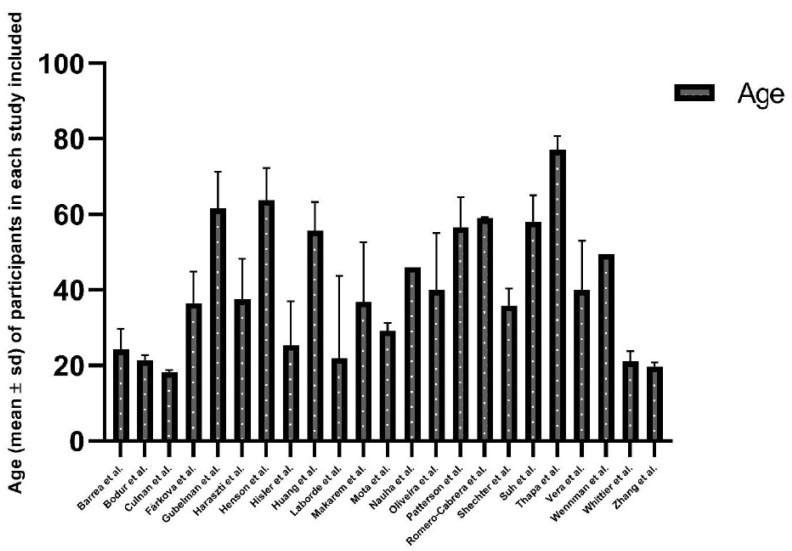
Age (mean) of participants in each included study [26,27,28,29,30,31,32,33,34,35,36,37,38,39,40,41,42,43,44,45,46,47,48].

From all the studies included in this review, the sample consisted of 505,375 participants and there was a great variability between the sample sizes ranging from 22 subjects [38] to 439,933 participants [37], with an average size of 21,973 participants. However, 14 of the 23 selected studies included more than 500 participants and just 3 studies analysed less than 100 subjects. Regarding the age of the participants included in the study, the average age was 40.73 years, and the mean ages ranged from 18.25 years [27] to 77 years [40] as it is shown in Figure 2.

Some of the studies focused on specific populations, such as women with polycystic ovary syndrome [44], participants with obesity or overweight [45,48], type 2 diabetes [46], prior cardiovascular disease [47] or adults during a COVID-19 lockdown [36]. A total of 4 out the 23 studies are focused on university students with a mean age below 25 years old [26,27,42,43]. Concerning the design of the included studies, most of the publications were cross-sectional observational studies and only five were longitudinal studies [27,31,35,45,47].

### 3.2. Chronotype and PA Collection Instruments

The most commonly used measurement to assess chronotype information is the 19-item Morningness–Eveningness Questionnaire (MEQ) validated by Horne and Ostberg [6] and implemented in 14 of the 23 included studies. MEQ is a questionnaire that includes items on sleep habits and fatigue and assesses individual differences in the degree to which respondents are active and alert at certain times of day. The scale item responses determine preferences in sleep and waking times and preferences for performing specific tasks during the day and at which respondents feel their best. Scores range from 16 to 86: lower scores indicate eveningness and higher scores indicate morningness. Individuals were classified as either evening type (score of ≤52), intermediate type (53–64) or morning type (≥65). Two different short versions of MEQ including 5 questions [49] or 6 questions [50] were used by three more studies [27,35,41] in order to classify participants in MC, IC or EC according to the total score.

Some different subjective questionnaires such as the Munich Chronotype Questionnaire (MCTQ) [45], the Caen Chronotype Questionnaire [32] or the Composite Scale of Morningness [29,30] were also employed in some included studies. The MCTQ is a self-report instrument that contains 29 questions about time of waking up and falling asleep on workdays and on free days separately [5,51]. This questionnaire is not based only on subjective sleep preferences, as it incorporates actual sleep behaviour collected information about when respondents prepare for sleep and go to bed, how long they sleep, when they wake up and when they get out of bed on work and non-work days. It quantifies in hours and minutes the discrepancy between free and working days (social jet-lag) and it has been used to control actigraphy interdaily stability in Farkova et al. [45]. Nevertheless, previous studies found that the continuous measures of MEQ score and MSFsc (corrected midpoint of sleep on free days derived from the MCTQ) were correlated [52]. The Caen Chronotype Questionnaire [53] is a 16-item questionnaire including two dimensions: a morningness– eveningness dimension (e.g., ‘‘my work goes better in the afternoon than before noon’’) and an amplitude dimension (e.g., ‘‘there are moments in the day where I would prefer to avoid any work’’). The Composite Scale of Morningness [54] includes 13 Likert-type questions compiling items from previous questionnaires in order to assess diurnal preference. Summed scores on the scale range from 13, indicating extreme eveningness, to 55, indicating extreme morningness. With the exception of the MCTQ, which measures the extent to which rhythmic biobehavioural events correspond to environmental ones, the other questionnaires evaluate similar concepts of sleep-wake cycles [55]. Additionally, other studies collected chronotypes using one self-reported question [36,37] or a combined variable for sleep patterns including the morning chronotype in healthy sleep habits [31].

On the other hand, three studies [38,45,47] collected, besides MEQ as a subjective measurement of chronotype, an objective measurement using actigraphy devices. Accelerometers make it possible to measure bedtime and wake time determined from sleep–wake logs. The midpoint of the sleep episode could be calculated as wake time minus half the total time in bed [38]. Likewise, acrophase was estimated considering the time period in a cycle during which the cycle crests or peaks, whereas the variable gets its maximum value. The lower the value of the acrophase, the earlier the peak activity. In order to estimate the objective chronotype, Fárkova et al. [45] analysed acrophase of activity collected by actigraphy, whereas Romero-Cabrera et al. [47] employed acrophase of an integrative variable combining temperature, activity and position.

Regarding the instruments to collect the PA, the included articles used different tools (objective or subjective) for PA information collection. A total of 7 out of the 23 studies employed objective (direct) methods. Meanwhile, 15 used subjective methods through questionnaires or self-reported variables, either exclusively or together with objective methods.

The International Physical Activity Questionnaire (IPAQ) [56] is a self-reported questionnaire to assess PA levels over the past week and it was used to collect PA in five studies [27,30,31,33,48] in its long version or a short form. Other included studies collected self-reported PA using other questionnaires, such as the Baecke questionnaire [34], the Minnesota Leisure Time Activity Questionnaire [47] or other self-reported questionnaires [26,29,36,37,39,41,42,43,44]. In some studies, the PA has been collected with just one question recall [42,43,44] or participation in sports [32]. On the other hand, some studies collected PA using objective measurement through accelerometers [28,30,35,36,38,45,46]. Just one study collected PA information combining both objective and self-reported instruments [30]. 

Sedentary behaviour (SB) was collected also through self-reported or direct methods using accelerometers [28,38,46,47]. Self-reported instruments cover a diversity or questionnaires about habitual minutes/day using a computer or watching TV [37], sitting duration [48], occupational SB, TV, computer, vehicle sitting or sitting elsewhere [41], physical inactivity [43] or a sedentary behaviour questionnaire [33].

### 3.3. Association between Chronotype, PA and SB

The 23 original primary studies included in this systematic review analysed the association between chronotype and PA levels or SB time in different populations using different collection instruments for chronotype and PA.

Of the studies included in the systematic review, 18 out of 23 found a significant relationship between the evening chronotype and less PA levels or more SB. Regarding PA levels, EC was associated with less PA time [28,35,39,41,43,48], less PA frequency [29], less walking and lower moderate to vigorous physical activity (MVPA) [37] and had greater odds of not meeting PA guidelines compared to MC [33]. 

Some studies used MEQ score as a continuous variable finding that higher scores (morningness preferences) showed a significant association with PA levels [34,40], more leisure-time PA [34] and more MVPA [38]. Other studies found that a diurnal preference was positively correlated with self-reported exercise and Fitbit exercise frequency [30] and that the decrease in the total volume of PA was significantly associated with the increase in eveningness preference during the COVID-19 lockdown [36]. The studies using objectives measures for chronotype found that the later the bedtime the participants (but not EC) were less active [47] and it was associated with less light PA and MVPA [38]. 

Regarding the SB, all 11 studies assessing SB found statistically significant differences between chronotype and SB: more sedentary time in EC compared to MC. These studies, included in this systematic review, found that EC was associated with more SB and with more time sitting, both using self-reported PA [33,37,41,43,47,48] or objective measurements using actigraphy [28,38,46,47]. In addition, Huang et al. [31] analysed a combined variable for healthy or poor sleep patterns considering MC as a healthy sleep factor. Results of their longitudinal study showed that poor sleep at baseline was associated with physical inactivity at follow-up and vice versa. On the other hand, Nauha et al. [35] only found that association for men participants.

Only four studies did not find any association between chronotype and PA in the general population. Three of those studies were focused on university students [26,27,42]. Lastly, Laborde et al. [32] found that chronotype was unrelated to sports participation.

Similarly, the studies focused on participants with a specific medical condition, such as type 2 diabetes [46], PCOS [44], obesity [48] or prior cardiovascular disease [47], found that EC were less active compared to MC showing less regular exercise [44], less PA time [47,48] and lower MVPA [46]. In addition, EC was associated with more SB and time sitting [46,47,48]. On the other hand, only Farkova et al. [45] did not find a significant association between objective chronotype measurement (acrophase) and PA levels in women with a BMI > 25 involved in a weight loss program.

Regarding the association in a specific population like university students, two studies did not find a significant relationship between MEQ chronotypes and PA in healthy university students in Turkey [26] and Peru [42]. In the same line, Culnan et al. [27] showed that changes in PA during the first year of university of students did not differ by chronotype in their longitudinal study. On the other hand, two studies [30,43] found a significant relationship between chronotype and PA. Hilser et al. [30] found that diurnal preference (using a composite scale of morningness) was positively correlated with self-reported PA (IPAQ) and Fitbit exercise frequency in university members (students and faculty) in Iowa, USA. Zhang et al. [43] found that the late chronotypes were associated with more sedentary behaviours and less PA time in medical students in China.

## 4. Discussion

### 4.1. Main Findings

This systematic review includes 23 studies fulfilling the selection criteria that analyses the relationship between chronotype, PA and SB. Overall, the results of this review show that EC is associated with less PA and more time in sedentary activities. This occurs independently of the instruments used to collect information about chronotype and PA, or the geographical area where the study was implemented with a wide spectrum of sun time and light exposure.

To our knowledge, this is the first systematic review analysing PA and SB and its relationship with the different chronotype categories [6]. A previous systematic review has focused on specific concepts about chronotype and athletic performance [20], describing that MC show less fatigue and better athletic performance in the morning. In addition, a systematic review found a relationship between chronotype and heart rate variability and a better sleep quality [21].

Nevertheless, the association between chronotype and PA and SB in our systematic review appeared to be related to the age of the participants. Based on our results, the association between chronotype and PA could be mitigated in young populations. In fact, four of the five studies included in our review with a mean age of the participants below 24 years old [26,27,32,42] did not show a significant relationship, except for the study developed by Zhang et al. [43] in Chinese medical students observing that late chronotypes were associated with more SB and less PA time. The information about SB and PA in this study was self-reported by the participants using one question for moderate PA and one question for SB. 

Meanwhile, among the studies with a mean age of the participants over that age, only one study did not find the association between chronotype and PA [45]. In that study, Fárkova et al. analysed the relationship between acrophase and mesor of PA (proportional to the overall mean activity during 24 h) obtained both via actigraphy—and it was not found a statistically significant association between those two direct measurements. Nevertheless, subjective chronotype measurements for chronotype (MEQ and Munich Questionnaire) were collected, but the relationship between PA level and those variables was not analysed. Subjective chronotype data were only employed to control the objective actigraphy measurements (acrophase and interdaily stability of the circadian rhythm). On the contrary, a relationship between PA and the stability of the circadian rhythm was observed.

Some studies included in this systematic review incorporated both direct and self-reported instruments in order to estimate the chronotype. In line with previous studies [9,57,58], objective and subjective measurements of chronotype were correlated. Thus, a later chronotype, reflected by a lower score on MEQ, was associated with a later bedtime, wake time and midpoint of sleep [38]. Likewise, EC according to MEQ showed delayed patterns of objective circadian rhythm and later acrophase both using activity [45] or a combined variable TAP [47].

Nevertheless, according to the previous literature, both approaches, even correlated, capture different characteristics related to chronotype. Subjective chronotype (based on questionnaires collecting timing preferences) may be reflective of underlying circadian physiology, whereas sleep timing (direct measurement through actigraphy) is a behaviour influenced by societal demands more dependent on willingness. In our review, both different variables were correlated and the association between PA and chronotype is shown in those articles [38,47] using either MEQ or direct data from actigraphy. Only one study [45] did not find a relationship between acrophase and PA. Nevertheless, a relationship between PA and stability of the circadian rhythm (obtained via actigraphy) was observed. Thus, regardless of chronotype, women with a stable circadian rhythm were significantly more active than women who did not have a stable one. According to their results, it seems relevant to keep a stable rhythm, independently of morningness or eveningness, living in accordance with one’s self-circadian phenotype. Further research is needed to confirm the association between PA and stable circadian rhythms.

### 4.2. Limitations of the Systematic Review

The present review was conducted following the PRISMA checklist. Despite this, one of the limitations of this study was the heterogeneity of the tools for the chronotype and PA assessment, sample size and characteristics of the population. At the same time, the consistency of the results, even with this heterogeneity, could reflect the magnitude of the impact of the chronotype on PA levels. Moreover, there are other constraints. First, the low number of articles showing results disaggregated by age and gender population groups. In addition, regarding the study design, most of the publications are cross-sectional studies; nevertheless, given the characteristics of the research question, it was expected that most of the designs would be observational.

### 4.3. Practical Implications and Future Research

The results of this systematic review confirm the existent association between chronotypes, PA and SB. It has direct implications for designing and implementing policies for PA promotion as well as for research studies analysing sleep-related variables and their relationship with lifestyles variables. Thus, regarding interventions focusing on increasing PA, it seems relevant to adjust the designs and the timing of the proposal according to the different chronotypes. A chronotype should be perceived as one of the barriers for users to join in physical activities. It may also be useful to implement PA maintenance strategies in EC with a tendency to be less active. This includes initiatives based on re-scheduling of academic or professional timing in order to encourage opportunities to perform PA despite the characteristics of the chronotype. It can also be useful to incorporate specific and individualized education strategies to promote PA according to the chronotype. The results may also be useful for the interpretation of PA assessments in clinical studies. Moreover, it is relevant to incorporate chronotype measurements in research studies assessing sleep routines, PA and SB as well as other lifestyle behaviours.

The relationship between chronotype and PA seems to not be so strong in younger adults and university students. Nevertheless, further research is needed in order to better understand the impact on those populations. The university period is a potentially appropriate stage to acquire healthy lifestyle habits due to the transition into autonomous life. In addition, college students are subjected to cyclic schedules (exam periods vs. class periods) and demanding social and academic situations. Furthermore, within the frame of healthy university strategies, it is relevant to incorporate the assessment of chronotype and circadian rhythm as well as health promotion strategies to chronotype-related issues.

## 5. Conclusions

Chronotypes are clearly associated with PA levels and SB, especially in populations over their mid-twenties. Evening chronotypes are associated with less PA and more time in sedentary activities compared to morning chronotypes. This occurs independently from the methods—objective or subjective—used to collect information about the chronotype and PA, geographical area or population with a specific medical condition.

Nevertheless, future studies are required to get more details about their impact on gender and different age ranges, especially college-aged populations. The impact of chronotypes should be considered in clinical research involving PA and is necessary for interventions on PA promotion. Specific strategies for evening chronotypes could be implemented in order to mitigate the potential impact on PA and other lifestyle behaviours.

## Figures and Tables

**Table 1:** Descriptive characteristics of the included studies on healthy population.

Author, Year	Country	Objective	Population	Chronotype Assessment	PA Assessment	Results
Bodur et al., 2021 [26]	Ankara, Turkey	To determine the role of sleep quality and caffeinated beverage consumption on the effect of late chronotype on body mass index (BMI)	*n* = 661 healthy university students Age (mean ± SD) 21.4 ± 1.38 years	MEQ	24 h PA record	No significant correlation was found between the chronotype scores and PA levels.
Culnan et al., 2013 [27]	USA	To test the relationship between chronotype in relation to BMI, energy expenditure and others	*n* = 137 colleges freshmen 79 females Age (mean ± SD) 18.25 ± 0.56	Short version MEQ	Changes in short-IPAQ	Changes in IPAQ did not differ by chronotype.
Gubelman et al., 2018 [28]	Lausanne, Switzerland.	To evaluate the association of objective PA and sedentary behaviour (SB) with sleep duration and quality	*n* = 2649 adults participating in CoLaus study. 53.5% women. Age (mean ± SD) 61.6 ± 9.8	MEQ	14 days accelerometer	High PA (RRR = 0.71; CI:0.52–0.97) and low sedentary behaviour (0.64; 0.47; 0.86) were significantly associated with lower likelihood of EC.
Haraszti et al., 2014 [29]	Budapest, Hungary	To explore the relationship between morningness–eveningness and perceived health	*n* = 202 female working at the university. Age (mean ± SD) = 37.5 ± 10.7 years	Composite scale of morningness	Adapted version of short-IPAQ	A significant lower frequency of physical exercise was associated with EC compared to MC (2.28 ± 1.5 vs. 2.85 ± 1.65 times per week; *p* = 0.042).
Hisler et al., 2017 [30]	Iowa, USA	To analyse if diurnal preference predicts variance in exercise frequency	*n* = 112 university members (students and faculty). 75% females Age (mean ± SD) 25.4 ±11.6	Composite scale of morningness	-FitBit -IPAQ	Diurnal preference (morningness) was positively correlated with self-reported exercise (r (105) = 0.36) and Fitbit exercise frequency (r (101) = 0.39).
Huang et al., 2021 [31]	UK	To analyse the association between sleep and PA	*n* = 38,601 UK Biobank participants (51% female, Age (mean ± SD) 55.7 ± 7.6 years	Combined sleep pattern variable	Weekly MET (IPAQ short-form) highly active, ≥1200; active, 600 to <1200; inactive <600)	Poor sleep pattern at baseline was associated with physical inactivity at follow-up (AOR = 1.65; 1.45–1.88) and vice versa.
Laborde et al., 2015 [32]	France	To explore how chronotype relates to various characteristics of sport training and competition	*n* = 976 non athletes + 974 athletes Women = 493 + 478 mean age 22.49 mean age: 21.21	Caen Chronotype Questionnaire	Sports participation	Morningness–eveningness was unrelated to sport participation
Makarem et al., 2020 [33]	USA	To evaluate associations of chronotype with overall cardiovascular health (CVH), health behaviours and cardiometabolic risk factors	*n* = 506 women participants of the GO Red study. Age (mean ± SD) = 37 ± 16 year	MEQ	IPAQ sedentary activities questionnaire	EC compared to MC was associated with greater odds of not meeting PA guidelines OR (95%CI) = 1.78 (1.03–3.07). Higher MEQ scores were also associated with significantly less sedentary time β (SE) = −0.11 (0.03).
Mota et al., 2016 [34]	Minas Gerais, Brazil	To analyse the association between chronotype, food intake and PA	*n* = 72 medical residents 52 women Age (mean ± SD) 29.2 ± 2.0	MEQ	Baecke questionnaire (BQ)	Chronotype score was positivity associated with leisure-time index (coefficient = 0.26, *p* = 0.03) and BQ total score (coefficient = 0.27, p = 0.03)
Nauha et al., 2020 [35]	Finland	To investigate an association between chronotype and objectively-measured PA and SED	*n* = 5156 participants Women: 2917 Age: 46 years	Short version MEQ	MET min/day (accelerometers) 14 days	Compared to EC, MC was associated with higher total (B;95%CI) (98.6; [30.2, 167.1] in men and in women (57.8; [10.5, 105.0]. Compared to EC, men with MC had less sedentary time(38.6; [−56.9, −20.2]).
Oliveira et al., 2021 [36]	Brazil	To investigate if PA changes might be associated with changes in the morningness–eveningness preference	*n* = 322 adults practicing social distancing during COVID-19 lockdown. 69% women Age (mean ± SD) 40 ± 15	Morningness–eveningness questionnaire score	Min/week self-reported questionnaire.	Decrease in the total volume of PA was significantly associated with the increase in eveningness preference. (3.7% *p* = 0.001) of the variance in the changes in MEQ score)
Patterson et al., 2016 [37]	UK	To examine the associations among sleep duration, chronotype and other variables	*n* = 439 933 participants in the UK Biobank project. 56% female Age (mean ± SD): 56.5 ± 8.1 years	One question self-reported chronotype questionnaire	-Self-reported minutes/week in walking, moderate and vigorous PA. -Self-reported minutes/day using a computer or TV on a typical day.	Early chronotypes reported accruing more mean minutes of walking (0.178; 0.011), moderate (0.172; 0.012) and vigorous activity (0.172; 0.017) and less screen based sedentary behaviour (0.313; 0.011) than late chronotypes (β; SE)
Shechter et al., 2014 [38]	USA	To determine if sleep timing and/or quality are related to PA levels.	*n* = 22 participants 6 females Age range: 30–45 year	-Bedtime and wake-up time and midpoint of sleep (accelerometer) -MEQ	Accelerometers 7–18 days. Sedentary <100 cpm, light PA 100–1951, MVPA >1952 cpm	Later bedtime, wake time and midpoint of sleep are all associated with more time spent in sedentary (*p* < 0.02) and less time spent in light PA (*p* < 0.05) and MVPA (*p* < 0.01). Higher MEQ had a significantly higher percentage of time in MVPA compared to those in the lower MEQ group (4.64% vs. 1.99%). No differences were observed in the low versus high MEQ score subgroups in percentage of time spent in sedentary or light PA.
Suh et al., 2016 [39]	Korea	To investigate health behaviours, health-related quality of life (HRQOL) and sleep among chronotypes in a community-based sample	*n* = 2976 participants of the Korean KoGes study. 83 + 828 + 535 men Age (mean ± SD): 58.02 years ±7.05	MEQ	METs: seven days PA Recall (retrospective self-reported)	EC were found to have significantly lower levels of PA (MET; SD = 14.54; 23.33) compared to MC (24.70; 30.41) *p* < 0.0001
Thapa et al., 2020 [40]	Korea	To examine the association between chronotype, daily PA and the estimated risk of dementia	*n* = 170 community dwelling over 70 102 women Age (mean ± SD): 77.0 years (±3.7 years)	MEQ	Daily PA (accelerometer)	Higher MEQ scores showed a higher volume of PA (r = 0.42, *p* < 0.005) for aged >75y and (r = 0.31, *p* < 0.05) for ≤75y.
Wennman et al., 2015 [41]	Finland	To operationalize chronotype using analysis for a 6-item scale derived from the original MEQ	*n* = 4904 participants aged 25–74 years	Short version MEQ	-Leisure time PA, commuting PA, domestic PA (self-reported questionnaire) -Sedentary behaviours: self-reported sitting.	Evening types and the “tired, more-evening type” had higher odds for none to very low (OR [95%CI] = 3.01 [2.00, 4.53] as well as low PA (1.47 [1.01–2.13]), as compared to “morning type”. Evening type was associated with higher odds for more time spent sitting, as compared to “morning type” (1.69 [1.19, 2.41]).
Whittier et al., 2014 [42]	Peru	To evaluate patterns of circadian preferences and daytime sleepiness, and to examine the association between the consumption of stimulant beverages and evening chronotype	*n* = 2581 undergraduate students Age (mean ± SD): 21.1 ± 2.7) 61% women	MEQ	Self-reported PA (yes/no)	PA was not significantly associated with chronotype status.
Zhang et al., 2018 [43]	China	To explore whether increased caffeinated drinks consumption and PA can mediate the relationship between late chronotype and BMI.	*n* = 616 medical students Age (mean ± SD): 19.7 ± 1.1 34.9% male	MEQ	-One question self-reported moderate PA -One question self-reported sedentary behaviour.	Late chronotypes were associated with more sedentary behaviours (B = −0.05, SE = 0.01, *p* < 0.001) and less PA time (B= 0.12, SE = 0.01, *p* < 0.001)

AOR: Adjusted Odds Ratio; EC: evening chronotype; IPAQ: international physical activity questionnaire; MC: morning chronotype; MEQ: morningness–eveningness questionnaire; MET: metabolic equivalent of tasks; MVPA: Moderate to Vigorous physical activity; PA: physical activity; RRR: Risk Relative Ratio; TAP: Temperature, activity and position; SE: standard error.

**Table 2:** Descriptive characteristics of the included studies on specific populations.

Author, Year	Country	Objective	Population	Chronotype Assessment	PA Assessment	Results
Barrea et al., 2022 [44]	Italy	To investigate if chronotype categories could be used as tool to screen healthy habits in women with PCOS	*n* = 112 Women with PCOS Age (mean ± SD) 24.21 ± 5.47 years	MEQ	At least 30 min per day of exercise (yes/no)	EC did less regular exercise (6.3%) compared to MC (64.5%) or neither (39.4%) *p* < 0.001.
Farkova et al., 2019 [45]	Czech Republic	To investigate the association between circadian phenotype and PA in a weight loss programme.	*n* = 75 women with BMI > 25 Age (mean ± SD) 36.5; SD 8.3	-MEQ -MCTQ -actigraphy (acrophase)	Actigraphy (mesor)	Parameters referring to the activity are not related to the acrophase.
Henson et al., 2020 [46]	Midlands, UK	To analyse association between chronotype and type 2 diabetes.	*n* = 635 participants with type 2 diabetes. 34.6% female Age (mean ± SD) = 63.8 ± 8.4 years,	MEQ	Accelerometer for 7 days	EC had higher sedentary time (28.7 min/day; 95% CI 8.6 to 48.3) and lower MVPA levels (–9.7; –14.9 to –4.6) compared to MCs. Also, later PA time for EC.
Romero-Cabrera et al., 2021 [47]	Spain	To explore whether individual chronotypes were associated with cardiometabolic risk in patients	*n* = 857 participants in Cordioprev study Age (mean ± SD): 59 ± 0.3 17.2% female	-MEQ -Actigraphy (acrophase of a combined variable TAP)	-Minnesota Leisure-Time PA questionnaire. -Sedentary behaviour	EC (MEQ and objective measures) were less active than MC (201 vs. 251 min/week; *p* = 0.01) and more sedentary (750 vs. 659 min/week *p* < 0.01) during the follow-up.
Vera et al., 2018 [48]	Spain	To study the relative contributions of genetics, lifestyle and circadian-related physiological characteristics in metabolic risk of evening chronotype	*n* = 2126 participants of the ONTIME study 1722 women Age (mean ± SD): 40 ± 13	MEQ	-IPAQ. -Self-reported sitting duration	EC engaged in less physically activity than MC (3230 ± 225 vs. 4283 ± 217; *p* = 0.001) and spent longer hours sitting per day (8.1 ± 0.1 vs. 7.6 ± 0.1; *p* = 0.001).

EC: evening chronotype; IPAQ: international physical activity questionnaire; MC: morning chronotype; MCTQ: Munich Chronotype Questionnaire; MEQ: morningness–eveningness questionnaire; MVPA: Moderate to Vigorous physical activity; PA: physical activity.

## Data Availability

Not applicable.

## Supplementary Materials

The following supporting information can be downloaded at: https://www.mdpi.com/article/10.3390/ijerph19159646/s1, Table S1: Systematic research, Table S2: Methodology quality assessment according to JIB checklist for cohort studies, Table S3: Methodology quality assessment according to JIB checklist for cross-sectional studies.
