# Supplementary material for: Association between Chronotype, Physical Activity and Sedentary Behaviour: A Systematic Review
Source: Int J Environ Res Public Health. 2022 Aug 5;19(15):9646. doi: 10.3390/ijerph19159646 (PMC9367887; doi:10.3390/ijerph19159646)
Supplement: Supplementary file 1 [file ijerph-19-09646-s001.zip › Table S1.pdf]

Table S1. Systematic search strategies

MEDLINE	"Exercise"[MeSH Terms] AND ("chronotype"[All Fields] OR "chronotyped"[All Fields] OR "chronotypes"[All Fields])
	(("chronotype"[All Fields] OR "chronotyped"[All Fields] OR "chronotypes"[All Fields]) AND ("exercise"[MeSH Terms] OR "exercise"[All Fields] OR ("physical"[All Fields] AND "activity"[All Fields]) OR "physical activity"[All Fields])) AND (y_10[Filter])
	(("chronotype"[All Fields] OR "chronotyped"[All Fields] OR "chronotypes"[All Fields]) AND ("prefer"[All Fields] OR "preferable"[All Fields] OR "preferably"[All Fields] OR "prefered"[All Fields] OR "preference"[All Fields] OR "preferences"[All Fields] OR "preferred"[All Fields] OR "preferring"[All Fields] OR "prefers"[All Fields]) AND ("exercise"[MeSH Terms] OR "exercise"[All Fields] OR ("physical"[All Fields] AND "activity"[All Fields]) OR "physical activity"[All Fields])) AND (y_10[Filter])
	(("acrophase"[All Fields] OR "acrophases"[All Fields]) AND ("chronotype"[All Fields] OR "chronotyped"[All Fields] OR "chronotypes"[All Fields]) AND ("exercise"[MeSH Terms] OR "exercise"[All Fields] OR ("physical"[All Fields] AND "activity"[All Fields]) OR "physical activity"[All Fields])) AND (y_10[Filter])
	(("chronotype"[All Fields] OR "chronotyped"[All Fields] OR "chronotypes"[All Fields]) AND ("sedentaries"[All Fields] OR "sedentariness"[All Fields] OR "sedentary"[All Fields])) AND (y_10[Filter])
	("morningness"[All Fields] AND ("exercise"[MeSH Terms] OR "exercise"[All Fields] OR ("physical"[All Fields] AND "activity"[All Fields]) OR "physical activity"[All Fields])) AND (y_10[Filter])
Trip DataBase	("chronotype" OR chronotype preference ") AND ("physical activity")
	("morningness") AND ("physical activity")
Psycinfo	("chronotype" OR chronotype preference ") AND ("physical activity")
	("morningness") AND ("physical activity")
Cochrane Library	("chronotype" OR chronotype preference ") AND ("physical activity")
	("morningness") AND ("physical activity")
