# Supplementary material for: Association between Chronotype, Physical Activity and Sedentary Behaviour: A Systematic Review
Source: Int J Environ Res Public Health. 2022 Aug 5;19(15):9646. doi: 10.3390/ijerph19159646 (PMC9367887; doi:10.3390/ijerph19159646)
Supplement: Supplementary file 1 [file ijerph-19-09646-s001.zip › Table S2.pdf]

Table S2. Methodology quality assessment according to JIB checklist for cross-sectional studies.

Author (year)	Were the criteria for inclusion in the sample clearly defined?	Were the study subjects and the setting described in detail?	Was the exposure measured in a valid and reliable way?	Were objective, standard criteria used for measurement of the condition?	Were confounding factors identified?	Were strategies to deal with confounding factors stated?	Were the outcomes measured in a valid and reliable way?	Was appropriate statistical analysis used?
Barrea et al.	Y	Y	Y	Y	Y	N	Y	Y
Bodur et al.	Y	Y	Y	Y	Y	Y	Y	Y
Gubelman et al.	Y	Y	Y	Y	Y	Y	Y	Y
Haraszki et al.	Y	Y	Y	Y	Y	N	Y	Y
Henson et al.	Y	Y	Y	Y	Y	N	Y	Y
Hisler et al.	Y	Y	Y	Y	N	N	Y	Y
Laborde et al.	Y	Y	Y	Y	Y	Y	Y	Y
Makarem et al.	Y	Y	Y	Y	Y	N	Y	Y
Mota et al.	Y	Y	Y	Y	Y	N	Y	Y
Oliveira et al.	Y	Y	Y	Y	Y	N	Y	Y
Patterson et al.	Y	Y	Y	Y	Y	N	Y	Y
Shechter et al.	Y	Y	Y	Y	Y	N	Y	Y
Suh et al.	Y	Y	Y	Y	N	N	Y	Y
Thapa et al.	Y	Y	Y	Y	Y	N	Y	Y
Vera et al.	N	Y	Y	Y	Y	N	Y	Y
Wennman et al.	Y	Y	Y	Y	Y	N	Y	Y
Whittier et al.	Y	Y	Y	Y	Y	N	Y	Y

Author (year)	Were the criteria for inclusion in the sample clearly defined?	Were the study subjects and the setting described in detail?	Was the exposure measured in a valid and reliable way?	Were objective, standard criteria used for measurement of the condition?	Were confounding factors identified?	Were strategies to deal with confounding factors stated?	Were the outcomes measured in a valid and reliable way?	Was appropriate statistical analysis used?
Zhang et al.	Y	Y	Y	Y	Y	Y	Y	Y

Y: YES, N: NO.
