# Supplementary material for: Association between Chronotype, Physical Activity and Sedentary Behaviour: A Systematic Review
Source: Int J Environ Res Public Health. 2022 Aug 5;19(15):9646. doi: 10.3390/ijerph19159646 (PMC9367887; doi:10.3390/ijerph19159646)
Supplement: Supplementary file 1 [file ijerph-19-09646-s001.zip › Table S3.pdf]

Table S3. Methodology quality assessment according to JIB checklist for cohort studies.

Author (year)	Were the two groups similar and recruited from the same population ?	Were the exposures measured similarly to assign people to both exposed and unexposed groups?	Was the exposure measured in a valid and reliable way?	Were confoundi ng factors identified?	Were strategies to deal with confoundi ng factors stated?	Were the groups/par ticipants free of the outcome at the start of the study (or at the moment of exposure)?	Were the outcomes measured in a valid and reliable way?	Was the follow up time reported and sufficient to be long enough for outcomes to occur?	Was follow up complete, and if not, were the reasons to loss to follow up described and explored?	Were strategies to address incomplete follow up utilized?	Was appropriat e statistical analysis used?
Culnan et al.	Y	Y	Y	N	N	Y	Y	NA	NA	Y	Y
Farkova et al.	Y	Y	Y	Y	Y	Y	Y	Y	N	N	Y
Huang et al.	Y	Y	Y	Y	Y	N	Y	NA	N	N	Y
Nauha et al.	Y	Y	Y	Y	Y	Y	Y	NA	NA	NA	Y
Romero-Cabrera et al.	Y	Y	Y	Y	Y	Y	Y	NA	NA	Y	Y

Y: Yes; N: No; NA: not-applicable
